# Human T-lymphotropic virus type 1 (HTLV-1) prevalence and quantitative detection of DNA proviral load in individuals with indeterminate/positive serological results

**DOI:** 10.1186/1471-2334-6-41

**Published:** 2006-03-02

**Authors:** Francesca Vitone, Davide Gibellini, Pasqua Schiavone, Antonietta D'Antuono, Lorenzo Gianni, Isabella Bon, Maria Carla Re

**Affiliations:** 1 Section of Microbiology, Department of Clinical and Experimental Medicine, University of Bologna, 40138 Bologna, Italy; 2 Dermatology, Department of Clinical and Experimental Medicine, University of Bologna, 40138 Bologna, Italy; 3 Oncology Division, Ospedale Infermi, 47900 Rimini, Italy

## Abstract

**Background:**

HTLV-1 infection is currently restricted to endemic areas. To define the prevalence of HTLV-1 infection in patients living in Italy, we first carried out a retrospective serological analysis in a group of people originating from African countries referred to our hospital from January 2003 to February 2005. We subsequently applied a real time PCR on peripheral blood mononuclear cells from subjects with positive or indeterminate serological results.

**Methods:**

All the sera were first analysed by serological methods (ELISA and/or Western Blotting) and then the peripheral blood mononuclear cells from subjects with positive or inconclusive serological results were analyzed for the presence of proviral DNA by a sensitive SYBR Green real time PCR. In addition, twenty HTLV-I ELISA negative samples were assayed by real time PCR approach as negative controls.

**Results:**

Serological results disclosed serum reactivity by ELISA (absorbance values equal or greater than the cut-off value) in 9 out of 3408 individuals attending the Sexually Transmitted Diseases Clinic and/or Oncology Department, and 2 out 534 blood donors enrolled as a control population. Irrespective of positive or inconclusive serological results, all these subjects were analyzed for the presence of proviral DNA in peripheral blood mononuclear cells by SYBR real time PCR. A clear-cut positive result for the presence of HTLV-1 DNA was obtained in two subjects from endemic areas.

**Conclusion:**

SYBR real time PCR cut short inconclusive serological results. This rapid and inexpensive assay showed an excellent linear dynamic range, specificity and reproducibility readily revealing and quantifying the presence of virus in PBMCs. Our results highlight the need to monitor the presence of HTLV-1 in countries which have seen a large influx of immigrants in recent years. Epidemiological surveillance and correct diagnosis are recommended to verify the prevalence and incidence of a new undesirable phenomenon.

## Background

HTLV-1 (Human T-cell lymphotropic virus type 1) is etiologically linked with adult T-cell leukemia (ATL) [1-3]. HTLV-I infection is geographically confined in specific areas such as Japan, the Caribbean basin, South America, Sub-Saharian Africa, Melanesia and the Middle East [4]. Japanese area-related studies estimated about one million people are currently infected by HTLV-I with 1–5% of infected patients showing developing ATL [5]. Therefore, the majority of HTLV-I infected subjects remain asymptomatic throughout their lives even though up to7% of HTLV-1 carriers may show chronic inflammatory neurological disease represented by HTLV-I- associated myelopathy/tropical spastic paraparesis (HAM/TSP) [1,3,6-9]. The relative percentage of malignant lymphoid proliferation and other associated diseases (such as HAM, uveites, poliomiosites, arthritis, and alveolitis) varies widely in the Caucasian population [4,10]. Hence, the transmission routes (such as sexual intercourse, blood transfusion, tissue transplantation and prolonged breastfeeding) [11-14] and the increasing number of individuals emigrated from endemic areas suggest that blood and tissue donors should be screened to reduce the spread of infection [15-17]. However, the epidemiology of HTLV-1 infection could change in the near future [18] in the wake of immigration. European countries, Italy particularly, represent the main destination for immigrants from the Middle East and Africa, making epidemiological surveillance highly recommended to ascertain the prevalence and incidence of HTLV-1 infection [16,19,20]. In the recent years, a number of countries, including USA, Canada and France, have introduced screening for blood donors to avoid a possible spread of HTLV-1 infection by blood transfusion [21].

To date, blood screening for HTLV-I has not been mandatory in Italy, but a more careful screening of the population might be justified by several literature reports [22-24]. Screening tests are usually based on antibody detection by ELISA and western blot, even though the relatively large number of indeterminate results (up to 2.5%) [21,25] needs to be confirmed by highly sensitive molecular techniques [14,22]. In addition, to establish the presence of the genome and its modulation over time and/or in the presence of specific therapy, PCR methods (commercially available and in-house modified tests) represent the gold standard useful to obtain a high level of specificity and reproducibility in a short time [17,26-28].

Considering the need to update information on HTLV-1 incidence in Italy, we investigated the presence of HTLV-1 infection in a selected group of patients originating from endemic areas using serological methods and a SYBR Green real time PCR technique able to verify and quantify the HTLV-1 proviral load.

## Methods

### Patients

From January 2003 to February 2005 we enrolled in the study a group of HIV-1/2 negative 3408 recent immigrants from African countries referred to the Sexually Transmitted Diseases Clinic and/or Oncology Department (group 1) and a group of 534 blood donors (group 2) undergoing laboratory analysis for serological diagnosis of other infectious diseases. Recent immigrants were defined as people stating they had lived in Italy for less than five years at the time of HTLV-I serological analysis. All patients, after informed consent, were screened for HTLV-1 antibodies by ELISA assay (Vironostika HTLV-I/II, BioMerieux, Boxtel, The Netherlands) as described by the manufacturer. In addition, all ELISA borderline or positive samples and some randomly selected ELISA negative samples were analyzed by Western-blot technique (Diagnostic Biotechnology HTLV WB, version 2.3, Genelabs Diagnostic, Singapore) following the manufacturers' procedure.

### Cell lines, plasmid and PBMC DNA extraction and purification

MT2 lymphoblastoid T cell line the most commonly used cell line for HTLV-I production, previously characterized as having 2.1 HTLV-I copies of virus/cell [29,30], Jurkat and 8E5LAV cells (a cell line carrying one copy of HIV-1 genome/cell) [31] were obtained from the American Type Culture Collection, (ATCC, Manassas, VA) and were kept in RPMI 1640 (Gibco, Grand Island, NY) supplemented with 10% of foetal calf serum (FCS; Gibco).

Peripheral blood mononuclear cells (PBMCs) from twenty HTLV-1 negative patients and eleven patients with indeterminate or positive HTLV-I serological assay were isolated from whole blood by Ficoll-HistoPaque gradient separation (Amersham Pharmacia). As previously described [32], DNA was extracted and purified from MT2, Jurkat, 8E5LAV and patients' PBMCs by DNAeasy tissue kit (Qiagen) following the manufacturer's instructions. The DNA content of each sample was determined by spectrophotometric analysis at 260/280 nm and stored at -80°C until use. PBMC, MT2, 8E5LAV and Jurkat cell pellets, corresponding to 5 × 10^6^, were prepared and stored at -80°C until use. Plasmid pHP1 is represented by the pCR2.1-TOPO vector (Invitrogen, Paisley, UK) where it was cloned the HTLV-I 117 bp amplicon as indicated by the manufacturer. The plasmid pHP1 was purified by Midi plasmid extraction kit (Qiagen) following the manufacturer's procedure. Genomic and plasmid DNA concentration and purity were determined by spectrophotometric analysis at 260/280 nm.

### Determination of HTLV-1 proviral DNA by SYBR green real time PCR

SYBR green real time PCR assay was performed in 20 μl PCR mixture volume consisting in 2× Quantitect SYBR green PCR Master Mix (Quiagen) containing HotStarTaq DNA polymerase, 200 nM of each oligonucleotide primer and 5 μl of DNA extracted from scalar dilution of MT-2 cell line (from 10^5 ^to 1 copies of HTLV-I genome) and clinical samples.

HTLV-1 *pol *gene amplification was carried out as follows: one cycle of 15 min at 95°C (hot-start PCR) and 45 cycles in four step each (95°C for 5s, 60°C for 30 s, 72°C for 30 s, 76°C for 5 s). At the end of amplification cycles, melting temperature was analyzed by a slow increase in temperature (0.1°C/s) up 95°C. The property elicited an accurate analysis of the melting temperature curve of the amplified fragments generated by real time PCR to determine the detection and quantification of specific products [33]. Real time target amplification profile demonstrated a specific main peak with a melting temperature at 79.35°C.

HTLV-I *pol *primer sequences were: 5' GTG GTG GAT TTG CCA TCG GGT TTT 3' and: 5' GTA CTT TAC TGA CAA ACC CGA CCT AC 3'. The amplification with this pair of oligonucleotides yielded a 117 bp *pol *fragment. Three replicates were done for each scalar dilution for intra-assay validation whereas three experiments were performed in triplicate for inter-assay analysis. All MT-2 DNA scalar dilutions were equalized at 600 ng of total DNA by Jurkat cell DNA addition in all experiments. In addition, 600 ng of total DNA from patients' PBMC or HTLV-I negative cell lines were amplified. All patients' samples were also analysed by SYBR Green real-time PCR for globin gene in a parallel run to check the equal amount in all samples determined by spectrophotometric data as described [34]. All standard dilutions, controls and samples from patients were run in duplicate and the average value of the copy number was used to quantify HIV-1 DNA copies in PBMC. All samples were run twice. The MT-2 cells were employed as reference curve when the clinical samples were assayed. Jurkat and 8E5LAV cell lines are employed as negative reference controls. HTLV-I DNA proviral load final quantitative data were expressed as number of copies per 10^6 ^PBMC. Exact values were used for calculations, ruling out decimal values.

Electrophoresis agarose gel, and Southern blot of PCR amplicons were carried out as previously described [33]. The hybridization was performed using a specific digoxigenin labelled oligonucleotide probe. The hybridization probe sequence is 5'-TAGCCCTATGGACAATCAAC -3'

## Results

### Serological analysis of patients' sera for specific HTLV-I antibodies

**Figure 1:**
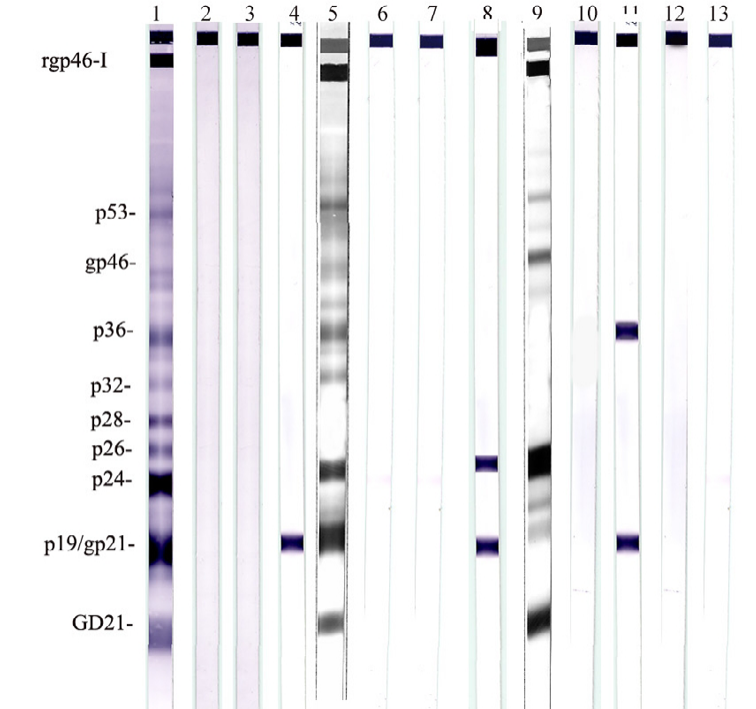
Western Blot analysis of samples with indeterminate or positive serological results. Lanes 1 and 2: control sera (positive and negative). Lanes 3 to 13 Western blot pattern of serum samples from patients 1 to 11 of table 1.

We analyzed the sera from 3408 immigrant African individuals HIV-1/2 negative, attending the Sexually Transmitted Diseases Clinic and/or Oncology Department (Group 1), and 534 blood donors by ELISA assay. Among the patients selected, serum reactivity (absorbance values equal or greater than the cut-off value) was disclosed in 9 out of 3408 patients (0.26%) and 2 out of 534 blood donors (Group 2). Hence, we assayed the reactive serum of these 11 individuals by HTLV-I specific Western-blot. As shown in Table 1 and in Figure 1, Western Blot analysis performed on these samples showed a clear serum reactivity to rgd21, p19, p24, p32, p36, gp46, p53 and rgp46-I proteins in patient n° 3 and a serum reactivity to rgd21, p19, p24, gp46, p53 and rgp46-I proteins in patient n° 7, both belonging to Group 1. Both samples (n° 3 and n° 7) were classified as positive on the basis of current guidelines [21]. In addition, a serum reactivity to p19 alone or in the presence of p26 or p30 was observed in three (patients n° 2, 6 and 9 of Group 1) out of the eleven and the absence of serum reactivity to any viral proteins in six (1, 4, 5, 8 of Group 1; 10 and 11 of Group 2) out of the eleven (Table 1).

**Table 1:** Western blot results in the eleven serum samples with different level of reactivity by immnoenzymatic assay (ELISA).

	WB results	ELISA results
Pt. n°1	negative	borderline
Pt. n°2	p19	borderline
Pt. n°3	gd21, p19, p24, p32, p36, gp46, p53, rgp46I	positive
Pt. n°4	negative	borderline
Pt. n°5	negative	borderline
Pt. n°6	p19, 26	borderline
Pt. n°7	gd21, p19, p24, gp46, p53 and rgp46-I	positive
Pt. n°8	negative	borderline
Pt. n°9	p19, 30	borderline
Pt. n°10	negative	borderline
Pt. n°11	negative	borderline

WB positive: serum reactivity to envelope and gag proteins.

WB indeterminate: serum reactivity to one or two HTLV-1 proteins.

WB negative: lack of reactivity to viral proteins.

ELISA borderline: absorbance value equal to or greater (ranging from 0.1 to 0.5 optical density) than the cut-off value

ELISA positive: absorbance value greater (ranging from 0.6 to >2.00 optical density) than the cut-off value

Western-blot assay results (Figure 1) demonstrated that only 2 samples out of 11 (18.2%) were confirmed serologically positive for HTLV-1 antibodies whereas 3 out of 11 (27.3%) were indeterminate and 6 out of 11 (54.5%) were negative.

In particular the two positive samples with a clear antibody pattern reacting to all HTLV-1 proteins belonged to patients originating from Nigeria (samples n°3) and Ghana (sample n°7) respectively. Interestingly, none of blood donors tested were Western-blot reactive or indeterminate for HTLV-I.

### SYBR Green-based real time PCR analysis of HTLV-I indeterminate and reactive Western-blot patients

We evaluated indeterminate and reactive Western-blot patients by in house SYBR green based Real time PCR technique to determine the presence of HTLV-I proviral genome in the peripheral blood mononuclear cells. This technique was validated on scalar dilutions (from 10^5 ^to 10 HTLV-I genome equivalent copies) of MT2 cell line genome containing 2.1 copies of proviral HTLV-I genomes/cell (29,30) by using an oligonucleotide specific pair able to amplify a 117 bp conserved region of HTLV-I *pol *gene. The assay encompasses at least five orders of magnitude with a high linear relationship (*r*^2 ^> 0.99) between the Ct values and the cell line input copies. The specificity of amplified products was assessed by melting curve analysis: all specific PCR amplicons showed the same dissociation temperature (79.35C°).

The sensitivity of SYBR Green real time PCR was assessed on MT-2 DNA scalar dilutions. The results demonstrated that repeated testing (three replicates of all scalar dilutions tested) of our assay disclosed 10 HTLV-I equivalent genome copies in 100% of replicates (Table 2), whereas a positive signal was not always detected when five copies were assayed in our experimental conditions.

**Table 2:** Intra-assay and inter-assay analysis of Ct mean value of standard curves obtained with scalar dilution of MT2 cell line (from 10^5 ^to 1).

**MT_2 _cell line N° copies/reaction**	**Ct mean values ± SD**	**CV**
**Intra-assay^a^**		

10^5^	23.35 ± 0.7	3.0
10^4^	25.91 ± 0.8	3.1
10^3^	28.10 ± 1.0	3.5
10^2^	31.78 ± 1.2	3.7
10	36.18 ± 1.3	3.5
1	ND	-
**Inter-assay^b^**		
10^5^	23.90 ± 0.7	2.9
10^4^	26.12 ± 0.9	3.4
10^3^	28.30 ± 1.1	3.8
10^2^	32.10 ± 1.3	4.0
10	36.50 ± 1.4	3.8
1	ND	-

SD : standard deviation; CV: coefficient of variation; ND: not detectable

^a ^For each sample, the Ct value is the average of results from three replicates.

^b ^For each sample, the Ct value is the average of results from three different experiments performed in triplicate.

**Figure 2:**
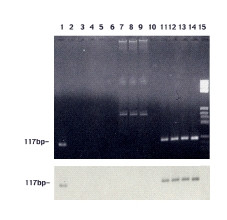
Agarose gel electrophoresis and Southern blot of samples amplified by SYBR Green real time PCR assay. In the upper part of the figure: the two positive HTLV-I samples (lanes 1 and 11), the samples with HTLV-I indeterminate or positive serological assays (lanes 2–10), MT-2 representative cells scalar dilutions (from 10^2 ^to 10^4; ^lanes 12–14) and molecular weight markers (lane 15) are shown. The bottom of the figure shows the Southern blot assay. The non-specific bands exhibited in the HTLV-I negative samples (lane 7–9) represent non-specific products that both did not hybridize with the HTLV-I specific internal probe and showed an unrelated melting temperature in SYBR Green real time PCR.

These data were confirmed when we analyzed the sensitivity of real time PCR by scalar dilutions of pHP1 plasmid where the HTLV-I 117 bp *pol *fragment was cloned in a plasmid vector. In this context, a real time PCR positive signal was always detected at 10 plasmid copies (data not shown). The specificity of SYBR Green real time PCR was determined by Jurkat or 8E5LAV cellular DNA analysis. These HTLV-I negative cells did not show any positive signal. Of note, this method shows a good intra- and inter- assay reproducibility determined by scalar dilutions of HTLV-I-positive MT-2 cell DNA equivalent genome analysis. In particular, *intra-assay *reproducibility was evaluated by three replicates of each point of scalar dilutions between 10^5 ^and 10 HTLV-I genome equivalent copies. The coefficient of variation (CV) of Ct was <3.8 % for all scalar dilutions tested. The CV of copy number was <35% for 10 copies and <25% for all more-concentrated dilutions (from 10^5 ^to 10^2 ^copy/sample) (Table 2). The *inter-assay *reproducibility was obtained by analysis of three different experiments, performed in triplicate, showing a CV for Ct <4.1% for all MT-2 DNA dilutions analysed. The CV of copy number was <40% for 10 copies and <25 % for all more-concentrated dilutions (Table 2). Hence, we applied this sensitive and specific technique on PBMCs DNA from the eleven subjects previously selected for Western-blot indeterminate or positive results. SYBR green based real time PCR technique disclosed the HTLV-I genome in the two Western-blot positive samples whereas the ELISA positive but Western blot indeterminate or negative samples did not display any significant positive fluorescent signal. In particular, the two real time PCR positive samples show a HTLV-I genome copy number of 1.2 × 10^5 ^(n°3 patient) and 5.9 × 10^2 ^(n°7 patient) per 10^6 ^PBMC genomes. Electrophoresis agarose gel and Southern blot assay analysis of amplicons indicated the presence of a specific band at 117 bp (Figure 2). An additional band representing an unspecific fragment was observed in some samples (lanes 7–9). This fragment did not hybridize with HTLV-I specific probe and displayed a higher melting temperature than HTLV-I specific products. In addition, no positive signal was found in samples from twenty individuals (randomly selected among the blood donors enrolled in the study) with negative ELISA results.

Oncologic clinical investigation of patient n°3 revealed that the positive sample belonged to a 27-year-old Nigeria-born female prostitute living in Italy since 2001. History-taking disclosed ATL disease with diffuse exfoliative dermatitis followed by a rapid deterioration of the patient's general condition, high white blood cell (WBC) count (total WBCs: 54,070/mm^3^), a high number of lymphocytes (35,684/mm^3^), severe hypercalcemia (12.7 mEq/L; normal values: 4.2–5.2 mEq/L) and elevated LDH plasma values (5,630 U/L; normal values: 230–450 U/L). Hematological findings showing a monoclonal T-cell lymphocytosis (95% of peripheral blood lymphocytes were CD3+/CD4+/CD8/TCR α/β) confirmed the diagnosis of ATL.

Patient n°7, a 29-year-old Ghana-born female prostitute living in Italy since 2000, monitored by the Sexually Transmitted Diseases Clinic, showed normal white blood cell count and number of lymphocytes, whereas no clinical information was accessible because the patient, when identified as HTLV-1 positive, refused any further control and/or hematological monitoring.

Neither patient displayed any serological reactivity for human immunodeficiency virus types 1 and 2, human hepatitis B virus or human hepatitis C virus infection.

## Discussion

The increasing rate of immigration towards European countries and global tourism has determined a new approach by national health committees to control the spread of some infectious diseases previously confined to specific endemic areas. In particular, this new global situation elicits European countries to monitor the local epidemiology of these emerging diseases. In Italy, HTLV-I infection is still sporadic and is confined to immigrants arriving from endemic areas. In particular, sexual transmission, HIV-1 seropositivity and intravenous drug abuse are the preferential infection routes that may lead an increase in HTLV-I infection incidence in the next few years. Our study, focused on disclosing the presence of HTLV-1 in Italy, revealed two HTLV-1 infections in a selected group of individuals originating from African countries. The specific presence of infection in Africa-born patients demonstrated that the infection is noticeable in individuals coming from endemic areas whereas no positive cases were found in the in Italy-born patient group [35-39]. These data are in accordance with several studies on HTLV-I epidemiology in Italy [10,18,19,40] even though a high prevalence of HTLV-I infection was more common in subjects co-infected with human immunodeficiency virus [19]. Our results disclosed HTLV-1 infection in two HIV-1 negative women living in Italy from several years. Even though we found a low prevalence (0.058%: two out the 3408 individuals enrolled in our study) of HTLV-1 infection, up-to-date information is necessary in non endemic countries to gain more knowledge on the spread of this virus.

In our study, both serological techniques used (ELISA and WB) revealed clear-cut positive results in two samples but Western blot did not rule out inconclusive results in three serum samples from high risk individuals. However, the finding of inconclusive results both by ELISA and immunoblotting analysis suggests the need to confirm virus presence by molecular methods. The identification of specific viral sequences in infected cells is essential to confirm the serological diagnosis in subjects with positive or indeterminate results [16,21,30]. In addition to determining the distribution of virus in the organism, amplification techniques also document the pathogenesis of infection and the effectiveness of antiviral therapy [41,42].

The clinical interest in molecular biology assays for HTLV diagnosis is increasing since proviral DNA levels represent a measure of integrated genome [43] and a surrogate marker of HTLV-I viral replication [44-46].

Our study also shows the application of a SYBR Green based real time PCR committed to HTLV-I provirus detection and quantitation. We optimised the conditions of SYBR Green real time PCR for HTLV-I DNA proviral detection with a high level of specificity (all 20 healthy blood donors' samples did not show any detectable fluorescent signal). Moreover, the assay has excellent dynamic range from 10^5 ^to 10^1 ^copies with a detection limit established at ten copies: a sensitivity comparable to other PCR formats for HTLV-I. SYBR Green was chosen instead of different real time approaches such as TaqMan or beacons to generate fluorescence signals, for several reasons. In particular, SYBR Green is less expensive than labelled probes that could also determine PCR artefacts beyond the 30^th ^cycle during the amplification. In addition, probe selected sequences may be prone to specific mutations [47].

The lack of any signal both in seronegative blood donors, used as negative controls and in samples with inconclusive serological results suggests a high specificity level. Some literature data suggest that an indeterminate serological profile (seroreactivity to env proteins, in particular to GD21) could reflect true HTLV-1 infection [16]. Interpretation of doubtful HTLV-1/2 Western blot patterns has therefore been enigmatic since the initiation of screening for these viruses in the late 1980s and several hypotheses have emerged in the last decade. Although HTLV-1 genomic sequences have been detected in the peripheral blood lymphocytes (PBL) of seropositive individuals, previous studies repeatedly demonstrated that PBL from the vast majority of HTLV-1/2 seroindeterminate individuals are PCR negative for HTLV-1 (48). However the possibility of a molecular biology technique characterized by an increased throughput over conventional PCR and quality performance must be taken into consideration for its wide application.

Since our SYBR green based real time PCR technique is a specific and simple assay for quantitative detection of HTLV-I proviral DNA, its application to monitor disease progression and verify the effectiveness of therapy offers an interesting option not only for first level diagnosis, but also for ongoing epidemiological surveillance.

HTLV-1 proviral DNA quantification opens interesting prospects. Cell-free viremia in plasma is not a prominent aspect of HTLV-associated diseases, unlike HIV infection, where the quantitative determination of RNA copies, the main prognostic parameter for disease evolution, directly mirrors viral replication [30]. Moreover, the determination of proviral load, in combination with other biomarkers, could be an important step in the pathogenesis of HTLV-associated disease. In particular, a significant correlation between the proviral load and neopterin concentration (related to inflammatory process in the spinal cord lesion) has been found in the CSF of HAM/TSP patients [44].

Our method may also be useful to analyze PBMCs from blood donors with serological indeterminate results. Blood donor screening for HTLV was introduced in Japan in the mid 1980s, in the United States and Canada in 1988 and finally in France in 1991 [21], and even though the probability of collecting blood products from a viremic donor is extremely low, it is not negligible.

## Conclusion

Even though our data demonstrated that HTLV-I infection is mainly confined to African immigrants, the feasibility of a simple and effective real time approach like our SYBR Green real time PCR suggests a possible application to cut short doubtful diagnosis of HTLV-1 infection and to monitor proviral and viral load during the course of infection and the efficacy antiviral therapy.

## Competing interests

The author(s) declare that they have no competing interests.

## Authors' contributions Section

MCR and DG conceived and designed the study. FV, PS and IB developed the HIV-1 DNA real time assay and performed all the experimental work. AD and LG provided blood samples and clinical information on the patients enrolled in this study. MCR drafted the manuscript and DG reviewed it. All authors contributed to the final version of manuscript, read and approved it.

## Pre-publication history

The pre-publication history for this paper can be accessed here:


